# Extra c-myc oncogene copies in high risk cutaneous malignant melanoma and melanoma metastases

**DOI:** 10.1054/bjoc.2000.1535

**Published:** 2001-01-01

**Authors:** G M Kraehn, J Utikal, M Udart, K M Greulich, G Bezold, P Kaskel, U Leiter, R U Peter

**Affiliations:** Department of Dermatology, University of Ulm, Ulm, Germany; Department of Pharmacology, University of California, La Jolla, San Diego, USA; Division of Carcinogenesis, Deutsches Krebsforschungszentrum (DKFZ), Heidelberg, Germany

**Keywords:** c-myc, chromosome 8, fluorescence in situ hybridization, RT-PCR

## Abstract

Amplification and overexpression of the c-myc gene have been associated with neoplastic transformation in a plethora of malignant tumours. We applied interphase fluorescence in situ hybridization (FISH) with a locus-specific probe for the c-myc gene (8q24) in combination with a corresponding chromosome 8 α-satellite probe to evaluate genetic alterations in 8 primary melanomas and 33 advanced melanomas and compared it to 12 melanocytic nevi, 7 safety margins and 2 cases of normal skin. Additionally, in metaphase spreads of 7 melanoma cell lines a whole chromosome 8 paint probe was used. We investigated the functionality of the c-myc gene by detecting c-myc RNA expression with RT-PCR and c-myc protein by immunohistochemistry. 4/8 primary melanomas and 11/33 melanoma metastases showed additional c-myc signals relative to the centromere of chromosome 8 copy number. None of the nevi, safety margins or normal skin samples demonstrated this gain. In 2/7 melanoma cell lines (C32 and WM 266–4) isochromosome 8q formation with a relative gain of c-myc copies and a loss of 8p was observed. The highest c-myc gene expression compared to GAPDH was found in melanoma metastases (17.5%). Nevi (6.6%) and primary melanomas (5.0%) expressed the c-myc gene on a lower level. 72.7% of the patients with c-myc extra copies had visceral melanoma metastases (UICC IV), patients without c-myc gain in 35.0% only. The collective with additional c-myc copies also expressed the gene on a significantly higher level. These results indicate that a c-myc gain in relation to the centromere 8 copy number might be associated with advanced cutaneous melanoma. © 2001 Cancer Research Campaign http://www.bjcancer.com


British Journal of Cancer (2001) 84(1), 72–79
© 2001 Cancer Research Campaign
doi: 10.1054/ bjoc.2000.1535, available online at http://www.idealibrary.com on IDEAL®

### Extra c-myc oncogene copies in high risk cutaneous malignant melanoma and melanoma metastases

GM Kraehn*[1,2], J Utikal*[1], M Udart[1], KM Greulich[1,3], G Bezold[1], P Kaskel[1], U Leiter[1] and RU Peter[1]

[1]Department of Dermatology, University of Ulm, Ulm, Germany; [2]Department of Pharmacology, University of California, San Diego, La Jolla, USA; [3]Division of Carcinogenesis, Deutsches Krebsforschungszentrum (DKFZ), Heidelberg, Germany

**Summary** Amplification and overexpression of the c-myc gene have been associated with neoplastic transformation in a plethora of malignant tumours. We applied interphase fluorescence in situ hybridization (FISH) with a locus-specific probe for the c-myc gene (8q24) in combination with a corresponding chromosome 8 $\alpha$-satellite probe to evaluate genetic alterations in 8 primary melanomas and 33 advanced melanomas and compared it to 12 melanocytic nevi, 7 safety margins and 2 cases of normal skin. Additionally, in metaphase spreads of 7 melanoma cell lines a whole chromosome 8 paint probe was used. We investigated the functionality of the c-myc gene by detecting c-myc RNA expression with RT-PCR and c-myc protein by immunohistochemistry. 4/8 primary melanomas and 11/33 melanoma metastases showed additional c-myc signals relative to the centromere of chromosome 8 copy number. None of the nevi, safety margins or normal skin samples demonstrated this gain. In 2/7 melanoma cell lines (C32 and WM 266–4) isochromosome 8q formation with a relative gain of c-myc copies and a loss of 8p was observed. The highest c-myc gene expression compared to GAPDH was found in melanoma metastases (17.5%). Nevi (6.6%) and primary melanomas (5.0%) expressed the c-myc gene on a lower level. 72.7% of the patients with c-myc extra copies had visceral melanoma metastases (UICC IV), patients without c-myc gain in 35.0% only. The collective with additional c-myc copies also expressed the gene on a significantly higher level. These results indicate that a c-myc gain in relation to the centromere 8 copy number might be associated with advanced cutaneous melanoma. © 2001 Cancer Research Campaign http://www.bjcancer.com

**Keywords**: c-myc; chromosome 8; fluorescence in situ hybridization; RT-PCR

The incidence of cutaneous melanoma has approximately doubled each decade for the last 50 years (Koh, 1991). It has been assumed that by the year 2000 one in 75 persons in the US will be affected by this type of skin cancer (Robinson et al, 1997). Cancer development is a complex, multistep process characterized by the abnormal activation of cellular proto-oncogenes and the loss of function of tumour suppressor genes (Vogelstein and Kinzler, 1993). A central role in the regulation of cell growth and differentiation is assigned to the c-myc proto-oncogene (Meichle et al, 1992). The c-myc gene is located to 8q24 (Taub et al, 1982) and it encodes a 439 amino acid phosphoprotein of 62 kDa with a predominantly nuclear localization (Cole, 1986). After the detection of an amplified c-myc gene locus in different human cell lines (Collins and Groudine, 1982; Little et al, 1983) oncogenic activation of the c-myc gene through amplification has been demonstrated. C-myc expression levels have been suggested to be of prognostic importance in several types of cancer (Garte, 1993). Contradictory results concerning the influence of c-myc expression on melanoma formation have been published, either arguing for a correlation of tumour progression with c-myc overexpression (Ross and Wilson, 1998) or for a lack of prognostic significance (Boni et al, 1998). Abnormalities of chromosome 8 have rarely been reported in cutaneous malignant melanoma (Pedersen et al, 1986; Ozisik et al, 1994). In contrast, additional copies of 8q are frequent in uveal melanoma (Horsman et al, 1990; Prescher et al, 1990), and the presence of additional 8q copies is associated with reduced survival (Sisley et al, 1997).

Interphase FISH (Cremer et al, 1986) is useful for the detection of numerical chromosomal abnormalities and genetic alterations in solid tumours including malignant melanomas, which are often difficult to analyse by conventional cytogenetics. In this study we enumerated and compared c-myc and chromosome 8 copy numbers within interphase nuclei (Hopman et al, 1994; Visscher et al, 1997) of normal skin, benign nevi, cutaneous primary and advanced malignant melanomas and corresponding safety margins.

Additionally, we analysed metaphase spreads from 7 melanoma cell lines with probes for c-myc, centromere of chromosome 8, and whole chromosome 8 (Pinkel et al, 1986; Lichter et al, 1991; Bar-Am et al, 1992).

### MATERIALS AND METHODS

62 cases including 8 primary cutaneous melanomas, 33 metastases of cutaneous malignant melanomas (13 lymph node and 20 distant cutaneous melanoma metastases), 12 nevi, 7 safety margins (surrounding macroscopically normal tissue between 1 and 3 cm from the margin of the primary tumours) and 2 samples of normal skin were collected from the Department of Dermatology at the University of Ulm, Germany. Touch preparations were prepared from unfixed tissue. Slides were fixed immediately in 4%

*Received 10 April 2000*
*Revised 24 August 2000*
*Accepted 25 August 2000*

*Correspondence to:* GM Kraehn

*G.M.K. and J.U. contributed equally to this work paraformaldehyde in 1 × PBS for 20 minutes, washed in 3× PBS, 1 × PBS, 1 × PBS (5 min each) and then dehydrated in ethanol (30%, 60%, 80%, 95%, 100%). Touch preparations were stained for S100 protein after standard protocols (DAKO, Germany) to confirm presence of tumour cells. In cases where cryoconserved tissue was available, the tumour material was chopped up and used for FISH cell preparations and simultaneously for isolation of RNA. The formalin-fixed parts of the tumours were sliced into 5 μm sections and stained for S100 and c-myc protein. Each slice was examined by two independent histopathologists.

### FISH

The method of interphase FISH was used as described elsewhere (Jenkins et al, 1997). Short time formalin fixed touch preparations of the specimens were incubated in 2 × SSC at 75˚C for 15 min, digested in pepsin solution (4 mg $ml^{-1}$ in 0.9% NaCl, pH 1.5) for 15 min at 37˚C, rinsed in 2× SSC at room temperature for 5 min and air dried. We used directly labelled fluorescent DNA probes (Vysis, Downers Grove, IL, USA) for the centromere region of chromosome 8 (CEP 8) and for the 8q24 (c-myc) region. Dual probe hybridization was performed on the pretreated touch preparations using a spectrum green labelled centromere 8 probe in combination with a spectrum orange labelled probe for c-myc gene. Probes and target DNA were denatured simultaneously in a 78˚C oven for 4 min and each slide was incubated at 42˚C overnight. Posthybridization washes were performed in 1.5 M Urea/0.1× SSC at 45˚C for 30 min and in 2 × SSC at room temperature for 2 min. Nuclei were counterstained with 4′,6-diamidino-2-phenylindole and antifade compound p-phenylenediamine. We used a fluorescence microscope (Zeiss, Germany) equipped with a digital camera connected to a computer with Mc Probe software (PSI, England) for analysis. Only signals in non-overlapping, apparently intact nuclei ($n$ = 100) were counted. An increased copy number for the centromere of chromosome 8 and the c-myc gene was defined as more than two signals in >10% of nuclei. Gain of the c-myc signal in relation to centromere 8 was defined as >25% of nuclei showing extra c-myc copies due to the fact that chromatid separation in proliferative cells results in an apparent increase in the number of signals located at distal parts of chromosomes (Hopman et al, 1994). The range of c-myc extra copies in relation to centromere 8 is indicated as a ratio calculated as x = c-myc signals per centromere 8 signals in all nuclei of the case. A c-myc/centromere 8 ratio of >1 indicates a relative c-myc gain if extra c-myc copies in relation to centromere 8 copy number are detected in >25% of nuclei.

### RNA isolation and c-myc RT-PCR

For RNA isolation the tissue was minced, homogenized, and RNA was isolated from homogenates using the RNA-Clean™ System (Angewandte Gentechnologie Systeme GmbH, Germany). Using oligo (dt) primers the extracted mRNA was transcribed to cDNA with a Reverse Transcriptase System (Promega, Madison, WI, USA) to cDNA. The cDNAs were then phenol/chloroform extracted, precipitated with ethanol and redissolved in $ddH_2O$. The cDNA concentration was measured by reading the absorbance at 260 nm. In PCR reactions (25 μl) 100 ng cDNA was used as template-DNA. For semiquantitative analysis of the expression level and to circumvent false negative results, we used primers detecting the GAPDH gene in the same reaction vessel. The PCR mixture contained, for each reaction, PCR-buffer, 1.5 mM $Mg^{2+}$, 0.2 mM each dNTP, 1.7 U Taq polymerase (Boehringer, Mannheim, Germany), 0.5 μM c-myc upstream primer (Stratagene, Heidelberg, Germany; 5′CCAGCAGCGACTCTGAGG-3′), 0.5 μM c-myc downstream primer (5′CCAAGACGTTGTG-TGTTC 3′), 0.5 μM GAPDH upstream primer (Stratagene 5′CCACCCATGGCAAATTCCATGGCA 3′), 0.5 μM GAPDH downstream primer (5′TCTAGACGGCAGGTCAGGTCCACC-3′) and 100 ng cDNA. PCR consisted of an initial denaturation step (94˚C 4 min), 30 amplification cycles (93˚C 35 sec, 60˚C 35 sec, 72˚C 35 sec) and a final extension step (68˚C 10 min). It was tested that PCR was still in the exponential phase at the end of cycling. The PCR products were visualized on a 2% agarose gel stained with ethidium bromide. Contamination of cDNAs with genomic DNA was ruled out by using intron-spanning primer sets. Densitometric analysis of c-myc and GAPDH bands was performed with a gel documentation system (Image Master VDS, Pharmacia, Freiburg, Germany). The PCR product was sequenced and the c-myc expression was calculated by setting the GAPDH expression as 100% and setting the c-myc expression in relation to this internal standard.

### Immunostaining of c-myc protein

4 μm sections were cut from paraffin blocks and were incubated in xylene and ethanol. The tissue sections were microwaved for 7 min in 0.01 M sodium citrate buffer, pH 6.0 and incubated with serum block for 20 min. Then sections were incubated with 2.5 μg/ml mouse anti-human c-myc monoclonal antibody (9E10, Santa Cruz, Heidelberg, Germany) for 2 hours at room temperature. Biotinylated secondary antibody was applied for 30 min. After washing in PBS, HRP-streptavidin complex was placed on the sections for 30 minutes and HRP substrate was added for 5 min. PBS instead of the primary antibody was used for negative controls. The intensity of cytoplasmic immunostaining was scored as negative, 1+, 2+ and 3+ for tumour cells corresponding to very pale staining, light brown staining and dark brown staining. As a positive control a melanoma tissue section known to express c-myc protein was immunostained.

### Cell lines

Metaphase spreads of the melanoma cell lines C 32, HT 144, RPMI 7951, SK MEL 2, SK MEL 28, SK MEL 31, WM 266–4 (American Type Culture Collection, Rockville, USA) were obtained by standard protocols. FISH with a whole chromosome painting (WCP) probe for chromosome 8, a centromere 8 and a c-myc probe (Vysis, Downers Grove, IL) was performed according to the manufacturer’s instructions. 20 metaphase spreads of each cell line were analysed and 100 interphase nuclei were counted for c-myc and centromere of chromosome 8 copies. RNA was isolated from each cell line at 3 different passages. Isolation of RNA and PCR were performed as described above.

### RESULTS

### Fluorescence in situ hybridization

FISH was performed on all 33 cutaneous melanoma metastases, 8 primary melanomas, 12 nevi, 7 safety margins, 2 specimen of normal skin from healthy donors and 7 melanoma cell lines.

© 2001 Cancer Research Campaign

**Table 1** Chromosome 8 and c-myc copies in melanoma cell lines

| Cell line no. | c-myc RNA expression | Nuclei with with extra c-myc copies | c-myc/ CEP 8 ratio | Probe | Percentage of nuclei with number of signals | | | | | | | | |
|---|---|---|---|---|---|---|---|---|---|---|---|---|---|
| | | | | | 0–1 | 2 | 3 | 4 | 5 | 6 | 7 | 8 | >9 |
| C 32 | 25.2 | 88% | 1.30 | c-myc | | | 18 | 32 | 32 | 12 | 4 | 2 | |
| | | | | CEP 8[a] | | 18 | 24 | 52 | 2 | 2 | 2 | | |
| WM 266–4 | 19.1 | 92% | 1.31 | c-myc | | | 2 | 88 | 8 | 1 | | 1 | |
| | | | | CEP 8 | | 3 | 84 | 11 | | 2 | | | |
| HT 144 | 15.8 | 1% | 0.95 | c-myc | | 3 | 73 | 18 | 1 | 5 | | | |
| | | | | CEP 8 | | 1 | 65 | 26 | 2 | 5 | 1 | | |
| RPMI 7951 | 6.8 | 4% | 0.95 | c-myc | | 2 | 60 | 32 | 4 | 2 | | | |
| | | | | CEP 8 | | | 50 | 44 | | 6 | | | |
| SK MEL 2 | 11.5 | 2% | 0.89 | c-myc | 18 | 54 | 12 | 16 | | | | | |
| | | | | CEP 8 | 2 | 70 | 2 | 26 | | | | | |
| SK MEL 28 | 18.5 | 2% | 1.00 | c-myc | | | 6 | 76 | 15 | 1 | | | 2 |
| | | | | CEP 8 | | | 4 | 73 | 21 | | | 1 | 1 |
| SK MEL 31 | 0 | 3% | 0.90 | c-myc | 1 | 11 | 36 | 30 | 15 | 3 | 1 | 1 | 2 |
| | | | | CEP 8 | | | 36 | 33 | 18 | 8 | 2 | 1 | 2 |

[a]Centromere of chromosome 8.

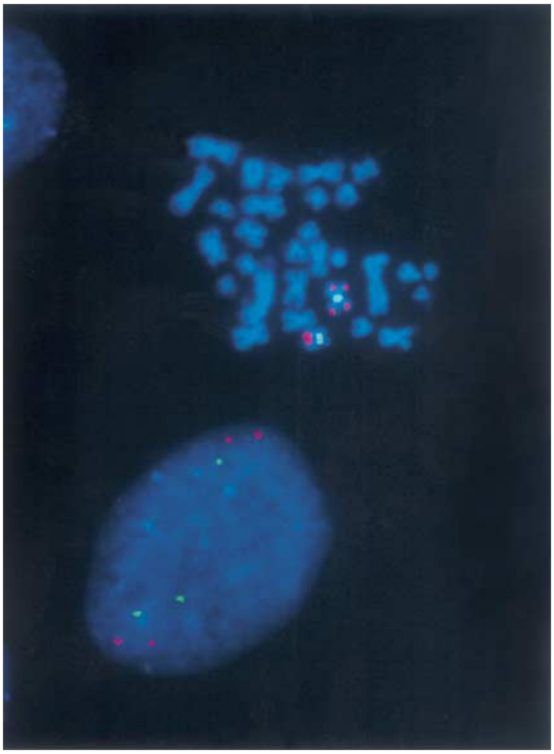

**Figure 1** Isochromosome 8q formation. The metaphase spread of the amelanotic melanoma cell line C32 shows isochromosome 8q formation with a relative gain of c-myc copies (orange signals: c-myc oncogene (8q24); green signals: centromere of chromosome 8). The interphase nucleus demonstrates 4 c-myc signals versus 3 centromere of chromosome 8 signals

### *Melanoma cell lines*

All 7 cell lines showed extra copies of c-myc oncogene in combination with the centromere 8 copy number detected by FISH (results are summarized in Table 1). Two cell lines (C 32 and WM 266–4) showed 8q isochromosome formation with a loss of 8p (Figure 1). Only one metaphase spread of the amelanotic melanoma cell line C 32 revealed an 8q isochromosome and an 8p isochromosome in addition to a normal chromosome 8 as detected by inverted DAPI-banding. In all 7 cases no chromosome 8 material was translocated to other chromosomes.

### *Benign controls, safety margins and normal skin*

As expected normal skin from healthy donors showed no signal gain. None of the 12 nevi showed extra copies of c-myc in relation to chromosome 8 copy number. Nuclei of 2 nevi demonstrated in 10% and 17% 3 respectively 4 chromosomes 8. Safety margins of cutaneous melanomas revealed no gain of chromosome 8 and c-myc copies in 4/6 specimens. One case was observed to have 3 chromosomes 8 in 10% nuclei. The second safety margin showed 2 signals for centromere 8 and 3 signals for c-myc gene locus in 14% of the nuclei.

### *Primary malignant melanomas*

The mean age of patients with primary melanoma was 71.8 years (median 72.5 years). 5 patients were male and 3 were female. 4 of the 8 primary malignant melanomas (see Table 2) were nodular malignant melanomas (NMMs) showing a high mean Breslow index of 7.61 mm (superficial spreading melanomas (SSMs) mean Breslow Index was 0.86 mm). All these NMM cases had a c-myc gain in relation to centromere 8 (Figure 2) while the other 4 cases which were different histological subtypes (1 lentigo maligna melanoma (LMM) and 3 SSMs) with lower tumour thickness showed no c-myc extra copies. NMMs had a c-myc/ centromere 8 ratio ranging from 1.20 to 2.84. Touch preparations of these NMMs demonstrated extra c-myc copies relative to the number of centromere 8 signals in 43%, 49%, 65% and 99% nuclei. Case 3 and 5 showed a c-myc increase in association with centromere 8 copy number.

### *Advanced malignant melanomas*

Patients with advanced malignant melanoma ranged in age from 17 to 87 years (mean 60.4 years; median, 59 years). The samples were collected from 17 women and 16 men. Of the 33 samples tested, 30 cases showed centromere 8 and c-myc gain. C-myc extra copies in relation to centromere 8 were found in 11 cases (4 lymph node metastases and 7 distant cutaneous metastases) (see

 *© 2001 Cancer Research Campaign*

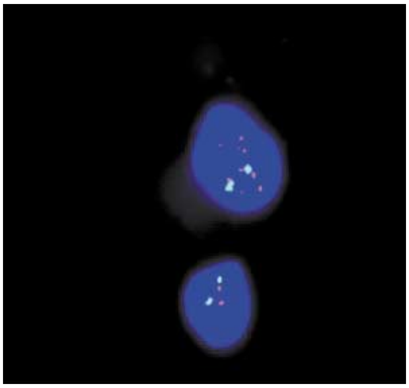

**Figure 2** C-myc amplification: Dual-probe FISH with a centromere 8 probe (green) and a region specific probe for c-myc gene (orange) showing a nucleus with c-myc extra copies in relation to centromere 8 and a normal nucleus in a touch preparation of a nodular malignant melanoma (case 8)

Table 3). C-myc/ centromere 8 ratio in those cases ranged from 1.22 to 2.90. Only case 26 showed one signal for centromere 8 with a c-myc gain (Figure 3) while the other specimens with c-myc extra copies had 2 and more signals for centromere 8. 3/11 (27.3%) patients with extra c-myc in relation to centromere 8 had cutaneous or lymph node metastases, whereas 8/11 (72.7%) patients revealed visceral metastases (including lung, bone, suprarenal, liver, peritoneal, spleen and cerebral metastases). In contrast to this collective, patients with balanced numbers of c-myc copies and centromere 8 had cutaneous or lymph node metastases in 65% (13/20) versus 35% (7/20) with visceral metastases (lung, cerebral, suprarenal, liver, bone) (see Figure 4). In both groups similar results for the Breslow index (mean 3.95 mm, median 1.85 mm with extra copies versus mean 2.76 mm, median 2.4 mm without additional c-myc copies) were found.

### RT-PCR

The amplified products of c-myc and GAPDH mRNA were detected as 345 bp and 598 bp fragments. 6/7 cell lines showed c-myc expression (data shown in Table 1). The highest c-myc RNA level in relation to GAPDH was found in those cell lines with isochromosome 8q formation (C32 mean 25.2% and WM 266–4 mean 19.1%). Melanoma metastases had the highest expression of the c-myc gene relative to GAPDH (mean 17.5%, median 16.9%). A similar level of c-myc mRNA was found in primary melanoma (mean 5.0%, median 2.15%) and in nevi (mean 6.62%, median 5.3%). In a single case of lentigo maligna a high c-myc expression comparable to those of metastases was detected (21.8%). Normal skin of healthy donors showed an average expression of 1.4%. Safety margins of melanomas had an expression of 4.2%. In one NMM (case no 8) a 3-fold excess could be detected in the tumour compared to the c-myc expression levels in the safety margin by RT-PCR. This tumour demonstrated additional c-myc gene copies in relation to the centromere 8 copy number.

### Immunostaining

To confirm the results on chromosomal and RNA level immunostaining with monoclonal antibody was performed on 25 sections. Nuclear and cytoplasmic staining for c-myc was found in 70% of all investigated melanoma metastases. An elevated c-myc expression was detected in those patients with the c-myc extra copies on the DNA level. In this group only one case was negative for c-myc staining. This group showed an average staining of 1.63+ (versus 0.66+ in the collective without additional c-myc copies). No significant difference of c-myc expression was found between nevi (53.8% positive) and primary cutaneous melanomas (60.0% positive).

**Table 2** Chromosome 8 and c-myc copies in primary malignant melanomas

| Case no. | Diagnosis/tumour thickness | c-myc RNA-expression | Nuclei with extra c-myc copies | c-myc/ CEP 8 ratio | Probe | Percent of nuclei with number of signals | | | | | | | | |
|---|---|---|---|---|---|---|---|---|---|---|---|---|---|---|
| | | | | | | 0–1 | 2 | 3 | 4 | 5 | 6 | 7 | 8 | >9 |
| 1 | SSM 0.8 mm | 2.0 | 8% | 0.92 | c-myc | 16 | 72 | 6 | 4 | | 2 | | | |
| | | | | | CEP 8[a] | 4 | 88 | 4 | 2 | 2 | | | | |
| 2 | SSM 0.75 mm | | 1% | 1.03 | c-myc | 5 | 87 | 2 | 6 | | | | | |
| | | | | | CEP 8 | 8 | 81 | 5 | 6 | | | | | |
| 3 | SSM 1.04 mm | 4.0 | 1% | 0.93 | c-myc | 3 | 80 | 15 | 2 | | | | | |
| | | | | | CEP 8 | 3 | 67 | 25 | 4 | | 1 | | | |
| 4 | NMM 11.0 mm | 14.7 | 49% | 1.30 | c-myc | 2 | 49 | 29 | 12 | 6 | 1 | | 1 | |
| | | | | | CEP 8 | 1 | 90 | 4 | 5 | | | | | |
| 5 | LMM not determined | | 9% | 0.95 | c-myc | 7 | 32 | 31 | 27 | 2 | 1 | | | |
| | | | | | CEP 8 | 3 | 45 | 10 | 36 | 4 | 1 | | 1 | |
| 6 | NMM 2.1 mm | | 43% | 1.43 | c-myc | 2 | 39 | 25 | 17 | 7 | 7 | 1 | 2 | |
| | | | | | CEP 8 | 4 | 78 | 7 | 11 | | | | | |
| 7 | NMM 12.1 mm | 2.0 | 65% | 1.20 | c-myc | 2 | 12 | 6 | 7 | 20 | 24 | 22 | 7 | |
| | | | | | CEP 8 | 1 | 14 | 10 | 16 | 38 | 19 | 2 | | |
| 8 | NMM 5.25 mm | 2.3 | 99% | 2.84 | c-myc | | 1 | | 18 | 28 | 28 | 17 | 6 | 2 |
| | | | | | CEP 8 | 2 | 96 | 2 | | | | | | |

[a]Centromere of chromosome 8.

© 2001 Cancer Research Campaign

**Table 3** Chromosome 8 and c-myc copies in melanoma metastases

| Case no. | Diagnosis (primary tumour, tumour thickness) | c-myc RNA-expression | Nuclei with extra c-myc copies | c-myc/CEP 8 ratio | Probe | Percent of nuclei with number of signals | | | | | | | | |
|---|---|---|---|---|---|---|---|---|---|---|---|---|---|---|
| | | | | | | 0–1 | 2 | 3 | 4 | 5 | 6 | 7 | 8 | >9 |
| 9 | metastasis (MM[a], not determined) | | 4% | 0.92 | c-myc | 24 | 74 | 2 | | | | | | |
| | | | | | CEP 8[b] | 14 | 86 | | | | | | | |
| 10 | metastasis (NMM, 8.0 mm) | | 2% | 0.97 | c-myc | 12 | 82 | 4 | 2 | | | | | |
| | | | | | CEP 8 | 7 | 87 | 4 | 2 | | | | | |
| 11 | metastasis (NMM, 2.75 mm) | 5.0 | 6% | 0.95 | c-myc | 20 | 68 | 8 | 2 | 2 | | | | |
| | | | | | CEP 8 | 5 | 89 | 2 | 2 | 2 | | | | |
| 12 | metastasis (SSM, 5.0 mm) | 13.0 | 5% | 1.03 | c-myc | 1 | 95 | 4 | | | | | | |
| | | | | | CEP 8 | 3 | 96 | 1 | | | | | | |
| 13 | metastasis (NMM, 1.2 mm) | | 12% | 1.04 | c-myc | 10 | 80 | 8 | 2 | | | | | |
| | | | | | CEP 8 | 7 | 92 | 1 | | | | | | |
| 14 | metastasis (SSM, 0.96 mm) | | 10% | 1.09 | c-myc | 2 | 88 | 6 | 2 | 2 | | | | |
| | | | | | CEP 8 | 7 | 93 | | | | | | | |
| 15 | metastasis (MM, not determined) | | 6% | 0.97 | c-myc | 5 | 79 | 12 | 4 | | | | | |
| | | | | | CEP 8 | 2 | 83 | 9 | 6 | | | | | |
| 16 | metastasis (SSM, 2.76 mm) | | 16% | 1.11 | c-myc | 3 | 81 | 10 | 4 | 2 | | | | |
| | | | | | CEP 8 | 4 | 96 | | | | | | | |
| 17 | metastasis (NMM, 2.3 mm) | 22.3 | 12% | 1.06 | c-myc | 9 | 73 | 10 | 6 | | 2 | | | |
| | | | | | CEP 8 | 8 | 82 | 4 | 6 | | | | | |
| 18 | metastasis (MM on nevus) | | 28% | 1.23 | c-myc | 8 | 64 | 20 | 6 | | | | 2 | |
| | | | | | CEP 8 | 11 | 86 | 2 | 1 | | | | | |
| 19 | metastasis (MM on nevus) | | 29% | 1.24 | c-myc | 2 | 70 | 13 | 8 | 4 | 1 | 1 | | 1 |
| | | | | | CEP 8 | 3 | 93 | 1 | 2 | 1 | | | | |
| 20 | metastasis (LMM, 14.0 mm) | | 63% | 1.31 | c-myc | 3 | 67 | 11 | 8 | 7 | 2 | 1 | 1 | |
| | | | | | CEP 8 | 2 | 96 | 1 | 1 | | | | | |
| 21 | metastasis (SSM, 2.2 mm) | | 54% | 1.32 | c-myc | 4 | 40 | 46 | 6 | 2 | | 2 | | |
| | | | | | CEP 8 | 3 | 91 | 4 | 2 | | | | | |
| 22 | metastasis (NMM, 2.05 mm) | | 2% | 0.98 | c-myc | 1 | 16 | 81 | 2 | | | | | |
| | | | | | CEP 8 | | 13 | 85 | 2 | | | | | |
| 23 | metastasis (SSM, 1.69 mm) | | 21% | 1.13 | c-myc | 2 | 54 | 17 | 19 | 5 | 1 | 1 | | 1 |
| | | | | | CEP 8 | 3 | 72 | 5 | 17 | 1 | 1 | | | 1 |
| 24 | metastasis (SSM, 0.9 mm) | | 13% | 1.07 | c-myc | | 22 | 69 | 3 | 2 | 4 | | | |
| | | | | | CEP 8 | | 26 | 72 | 1 | 1 | | | | |
| 25 | metastasis (MM, not determined) | | 10% | 1.04 | c-myc | | 8 | 84 | 2 | 2 | 4 | | | |
| | | | | | CEP 8 | 1 | 14 | 79 | 2 | 2 | 2 | | | |
| 26 | metastasis (SSM, 1.5 mm) | | 91% | 2.90 | c-myc | 2 | 8 | 61 | 26 | 1 | 2 | | | |
| | | | | | CEP 8 | 91 | 8 | 1 | | | | | | |
| 27 | metastasis (ALM, not determined) | | 8% | 1.05 | c-myc | 1 | 8 | 71 | 13 | 1 | 6 | | | |
| | | | | | CEP 8 | | 15 | 72 | 8 | 2 | 3 | | | |
| 28 | metastasis (NMM, 1.5 mm) | 5.5 | 1% | 0.93 | c-myc | 2 | 24 | 30 | 36 | 4 | 2 | | 2 | |
| | | | | | CEP 8 | | 22 | 12 | 56 | 8 | 2 | | | |
| 29 | metastasis (ALM, 3.3 mm) | 18.4 | 10% | 1.01 | c-myc | | 10 | 43 | 44 | 2 | | 1 | | |
| | | | | | CEP 8 | 1 | 13 | 41 | 42 | 1 | 1 | 1 | | |
| 30 | metastasis (NMM, 3.3 mm) | 24.6 | 64% | 1.66 | c-myc | 2 | 27 | 25 | 26 | 13 | 5 | 1 | | 1 |
| | | | | | CEP 8 | 5 | 80 | 13 | 2 | | | | | |
| 31 | metastasis (MM, not determined) | 9.3 | 6% | 1.00 | c-myc | 1 | 23 | 10 | 58 | 8 | | | | |
| | | | | | CEP 8 | | 24 | 9 | 63 | 4 | | | | |
| 32 | metastasis (ALM, 0.74 mm) | 39.1 | 65% | 1.30 | c-myc | | 14 | 18 | 53 | 12 | | 1 | | 2 |
| | | | | | CEP 8 | | 21 | 70 | 7 | 1 | 1 | | | |
| 33 | metastasis (NMM, 3.5 mm) | | 21% | 1.05 | c-myc | 1 | 9 | 7 | 75 | 5 | 1 | | | 2 |
| | | | | | CEP 8 | | 10 | 22 | 64 | 2 | | | 2 | |
| 34 | metastasis (MM, not determined) | | 16% | 1.08 | c-myc | 1 | 9 | 10 | 60 | 8 | 2 | 8 | 2 | |
| | | | | | CEP 8 | 1 | 9 | 16 | 66 | 2 | 4 | 2 | | |
| 35 | metastasis (NMM, 2.4 mm) | | 13% | 1.05 | c-myc | 4 | 25 | 1 | 24 | 23 | 6 | 5 | 3 | 9 |
| | | | | | CEP 8 | 1 | 25 | 2 | 32 | 29 | 1 | 1 | 2 | 7 |
| 36 | metastasis (SSM, 1.4 mm) | | 47% | 1.22 | c-myc | 1 | 2 | 4 | 51 | 14 | 21 | 3 | 3 | 1 |
| | | | | | CEP 8 | | 4 | 11 | 83 | 1 | 1 | | | |
| 37 | metastasis (MM, not determined) | | 51% | 1.32 | c-myc | | 14 | 8 | 23 | 22 | 17 | 7 | 6 | 3 |
| | | | | | CEP 8 | 2 | 31 | 13 | 26 | 12 | 15 | 1 | | |
| 38 | metastasis (SSM, 3.15 mm) | | 17% | 0.98 | c-myc | 2 | 4 | 5 | 11 | 30 | 27 | 17 | 2 | 2 |
| | | | | | CEP 8 | | 5 | 2 | 11 | 28 | 47 | 4 | | 3 |
| 39 | metastasis (MM, not determined) | 15.9 | 8% | 0.94 | c-myc | 1 | 5 | 2 | 10 | 33 | 34 | 4 | 9 | 2 |
| | | | | | CEP 8 | 3 | 4 | 2 | 5 | 36 | 36 | 1 | 5 | 8 |
| 40 | metastasis (ALM, 7.36 mm) | | 79% | 1.42 | c-myc | | 1 | 3 | 10 | 20 | 21 | 18 | 8 | 19 |
| | | | | | CEP 8 | | 1 | 10 | 45 | 32 | 2 | 1 | 6 | 3 |
| 41 | metastasis (SSM, 1.13 mm) | 22.2 | 96% | 1.59 | c-myc | | 2 | | | | 4 | 17 | 65 | 12 |
| | | | | | CEP 8 | | 2 | 2 | 9 | 85 | 2 | | | |

[a]Malignant melanoma. [b]Centromere of chromosome 8.

*© 2001 Cancer Research Campaign*

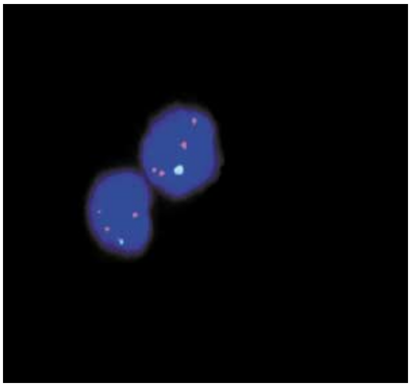

**Figure 3** Monosomie 8 and c-myc extra-copies. Melanoma metastasis with nuclei demonstrating one signal for centromere 8 and a relative gain for c-myc copies (case 26)

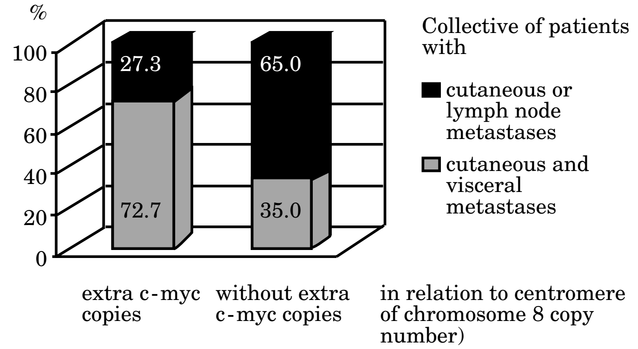

**Figure 4** Extra c-myc copies in patients with visceral metastases. Higher percentage of visceral metastases in the collective of patients with extra c-myc copies in relation to centromere 8 copy number in examined cutaneous metastases.

### DISCUSSION

Myc family oncoproteins have been demonstrated to be sequence-specific transcription factors that are believed to regulate the expression of genes governing cellular growth, differentiation, and programmed cell death (Chin et al, 1996; Thompson, 1998).

We report on c-myc gene extra copies and their relationship to centromere of chromosome 8 copy number in primary and advanced malignant melanoma in comparison to normal skin and benign nevi. One factor contributing to additional c-myc gene copies in tumour cells is non-polyploid gain of whole chromosome 8. Additional 8q arms or isochromosome 8q formation is another factor. Gain of 8q has been detected in posterior uveal melanoma by FISH (Sisley et al, 1997) and by CGH (Ghazvini et al, 1996). Our results of metaphase spreads of cutaneous melanoma cell lines showed isochromosome 8q formation in two cell lines. Thus we hypothesize that the observed intermediate relative increase in c-myc is caused by isochromes 8q. Homogeneously staining regions (hsr) and double minute chromosomes (dmin) are regarded as hallmarks of gene amplification which might be the basis for high c-myc expression (gene-dose-effect).

Furthermore, chromatid separation in highly proliferative cells results in an apparent intermediate increase in the number of c-myc signals while the number of centromere 8 signals remains constant during the cell cycle stages (Hopman et al, 1994). It may be estimated that c-myc signal gain in up to 25% of counted nuclei might reflect the proliferative activity.

C-myc oncogene extra copies related to centromere 8 in >25% of nuclei were found in 4/8 primary melanomas (Table 2) and in 11/33 melanoma metastases (Table 3). All benign control cases failed to show c-myc extra copies in relation to centromere 8. Our data suggest that additional c-myc gene copies and overexpression of c-myc – isolated or combined with other genes mapped to 8q – may play an important role in the progression of malignant melanoma. The high number of patients at an advanced stage of the disease with visceral metastases that had a c-myc copy number gain in relation to chromosome 8, in contrast to a relatively unaltered situation predominantly found in patients with cutaneous and lymph node metastases, is a striking result. A similar situation was found in SCID mice. Here, subcutaneous injection of a transfected melanoma cell line with high c-myc expression resulted in a more aggressive growth behaviour with lymph node and lung metastases compared to a cell line with lower c-myc expression (Schlagbauer-Wadl et al, 1999). Similar data exist in breast cancer where c-myc amplification is correlated with an aggressive clinical behaviour (Berns et al, 1992) or in colorectal cancer where c-myc amplification (Masramon et al, 1998) and c-myc mRNA expression assessed by RT-PCR (Kakisako et al, 1998) are indicative of a shorter disease-free survival.

In our cases the highest c-myc expression levels have been detected by RT-PCR in advanced melanomas, in contrast to relatively low levels in primary tumours and nevi. Our results both on RNA and protein level confirm published data showing similar c-myc expression on the protein level in primary melanoma and in nevi (Bergman et al, 1997) and a higher expression in melanoma metastases (Grover et al, 1996). Regarding the relation of c-myc expression to the number of c-myc FISH signals, nevi and a subset of primary tumours with a tumour thickness up to 1.04 mm have both a combination of low expression and low signal number, whereas primary melanomas with a high tumour thickness (5.2 mm; 11.0 mm; 12.1 mm) have either an elevated expression or a higher number of signals (Table 2, Figure 5). Metastases show an even more pronounced pattern: elevated expression, elevated gene copy number or a combination of both features (Figure 5). These

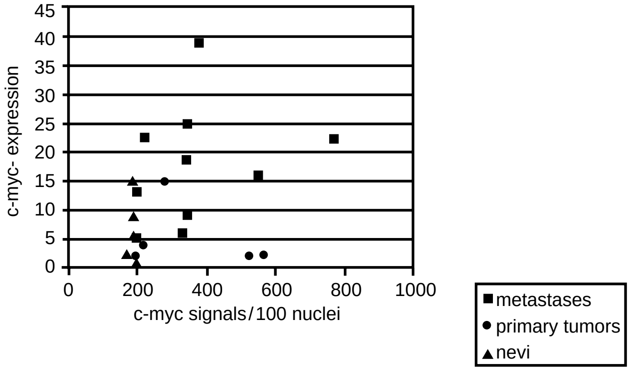

**Figure 5** C-myc expression levels/gene copies. C-myc expression in percentage of GAPDH expression and c-myc gene copy number in 100 nuclei of nevi, primary melanomas and metastases (cases where RNA was available)

© 2001 Cancer Research Campaign observations suggest a mechanism that involves transcription as well as polysomy in the progression of the disease.

From a clinical point of view 2 patients with metastases and additional c-myc copies had a low tumour thickness of the primary tumour (case 32: 0.74 mm and case 41: 1.13 mm). Based upon epidemiological data (Balch et al, 1985) this low tumour thickness would predict a benign course of the disease. The aggressive clinical behaviour of these two melanomas is in obvious contrast to the epidemiological data. C-myc extra copies might offer an explanation for this unexpected tumour progression in these 2 patients. C-myc copy number may also reflect a surrogate marker for genetic instability. The predominance of c-myc expression on RNA and protein level in metastases, associated with increased c-myc copy number in relation to centromere of chromosome 8 number, argues for the involvement of c-myc in late stage malignant melanoma. The data presented certainly warrant further prospective studies to elucidate the role of extra c-myc copies in relation to centromere 8 copy number as a genetic alteration reflecting the stage of the disease.

Findings of additional c-myc copies in combination with the centromere 8 copy number in one safety margin and 1 extra c-myc signal in relation to the centromere 8 copy number in 14% of nuclei in another safety margin is an interesting observation. This might be either caused by inapparent tumour cells or, more likely, by a high proliferative state of the normal tissue. Due to a two step excisional procedure of the primary melanoma fibrotic or inflammatory processes in the safety margin might result in a highly proliferative status, which could explain the alteration of the relative increase of the c-myc copy number in relation to the centromere 8 number. Alteration of gene expression and chromosomal aberration might help to determine the appropriate safety margin for melanoma in the future.

Additionally, based on the observation of down-modulation of c-myc expression by interferon γ in human melanoma cell lines (Osanto et al, 1992) and the enhancement of the efficacy of cisplatin in melanoma chemotherapy by c-myc antisense oligodeoxynucleotides both in vitro and in nude mice (Citro et al, 1998), c-myc expression in relation to chromosome 8 copy number might open new ways to identify patients with an increased chance to benefit from certain chemotherapeutic regimens.

### ACKNOWLEDGEMENTS

We thank Gerda Hack, Petra Miller and Christa Pantic for their excellent technical assistance. This work was partly supported by a grant from the Bundesministerium für Bildung, Forschung und Technologie (07 UV B 56/0).

### REFERENCES

Balch CM, Soong SJ, Shaw HM and Milton GW (1985) An analysis of prognostic factors in 4000 patients with cutaneous melanoma, In: Balch CM, Milton GW, Shaw HM, Soong SJ (eds) *Cutaneous melanoma. Clinical management and treatment results worldwide*, Lippincott: Philadelphia, pp 321–352

Bar-Am I, Mor O, Yeger H, Shiloh Y and Avivi L (1992) Detection of amplified DNA sequences in human tumor cell lines by fluorescence in situ hybridization. *Gene Chromosome Canc* **4**: 314–320

Bergman R, Lurie M, Kerner H, Kilim S and Friedman-Birnbaum R (1997) Mode of c-myc protein expression in Spitz nevi, common melanocytic nevi and malignant melanomas. *J Cutan Pathol* **24**: 219–222

Berns EM, Klijn JG, van Putten WL, van Staveren IL, Portengen H and Foekens JA (1992) C-myc amplification is a better prognostic factor than HER 2/ neu amplification in primary breast cancer. *Cancer Res* **52**: 1107–1113

Boni R, Bantschapp O, Muller B and Burg G (1998) C-myc is not useful as prognostic immunohistochemical marker in cutaneous melanoma. *Dermatology* **196**: 288–291

Chin L, Liégeois N, DePinho RA and Schreiber-Agus N (1996) Functional interactions among members of the myc superfamily and potential relevance to cutaneous growth and development. *J Invest Dermatol Symp Proc* **1**: 128–135

Citro G, D'Agnano I, Leonetti C, Perini R, Bucci B, Zon G, Calabretta B and Zupi G (1998) C-myc antisense oligodeoxynucleotides enhance the efficacy of cisplatin in melanoma chemotherapy in vitro and in nude mice. *Cancer Res* **58**: 283–289

Cole MD (1986) The myc oncogene: its role in transformation and differentiation. *Annu Rev Genet* **20**: 361–384

Collins S and Groudine M (1982) Amplification of endogenous myc-related DNA sequences in a human myeloid leukaemia cell line. *Nature* **298**: 679–681

Cremer T, Landegent J, Bruckner A et al (1986) Detection of chromosome aberrations in the human interphase nucleus by visualization of specific target DNAs with radioactive in situ hybridization techniques: diagnosis of trisomy 18 with probe L.1.84. *Hum Genet* **74**: 346–352

Garte SJ (1993) The c-myc oncogene in tumor progression. *Crit Rev Oncog* **4**: 435–449

Ghazvini S, Char DH, Kroll S, Waldman FM and Pinkel D (1996) Comparative genomic hybridization analysis of archival formalin fixed paraffin embedded uveal melanomas. *Cancer Genet Cytogen* **90**: 95–101

Grover R, Ross DA, Richman PI, Robinson B and Wilson GD (1996) C-myc oncogene expression in human melanoma and its relationship with tumour antigenicity. *Eur J Surg Oncol* **22**: 342–346

Hopman AH, Voorter CE and Ramaekers FC (1994) Detection of genomic changes in cancer by in situ hybridization. *Mol Biol Rep* **19**: 31–44

Horsman DE, Sroka H, Rootman J and White VA (1990) Monosomy 3 and isochromosome 8q in uveal melanoma. *Cancer Genet Cytogen* **45**: 249–253

Jenkins RB, Qian J, Lieber MM and Bostwick DG (1997) Detection of c-myc oncogene amplification and chromosomal anomalies in metastatic prostatic carcinoma by Fluorescence in Situ Hybridization. *Cancer Res* **57**: 524–531

Kakisako K, Miyahara M, Uchino S, Adachi Y and Kitano S (1998) Prognostic significance of c-myc mRNA expression assessed by semi-quantitative RT-PCR in patients with colorectal cancer. *Oncol Rep* **5**: 441–445

Koh HK (1991) Cutaneous melanoma. *N Engl J Med* **325**: 171–182

Lichter P, Boyle AL, Cremer T and Ward DC (1991) Analysis of genes and chromosomes by nonisotopic in situ hybridization. *GATA* **8**: 24–35

Little CD, Nau MM, Carney DN, Gazdar AF and Minna JD (1983) Amplification and expression of the c-myc oncogene in human lung cancer cell lines. *Nature* **306**: 194–196

Masramon L, Arribas R, Tortola S, Perucho M and Peinado MA (1998) Moderate amplifications of the c-myc gene correlate with molecular and clinicopathological parameters in colorectal cancer. *Brit J Cancer* **99**: 2349–2356

Meichle A, Philipp A and Eilers M (1992) The functions of Myc proteins. *Biochim Biophys Acta* **1114**: 129–146

Osanto S, Jansen R and Vloemans M (1992) Downmodulation of c-myc expression by interferon γ and tumour necrosis factor α precedes growth arrest in human melanoma cells. *Eur J Cancer* **28**A: 1622–1627

Ozisik YY, Meloni AM, Altungoz O, Peier A, Karakousis C, Leong SPL and Sandberg AA (1994) Cytogenetic findings in 21 malignant melanomas. *Cancer Genet Cytogen* **77**: 69–73

Pedersen MI, Bennett JW and Wang N (1986) Nonrandom chromosome structural aberrations and oncogene loci in human malignant melanoma. *Cancer Genet Cytogen* **20**: 11–17

Pinkel D, Straume T and Gray JW (1986) Cytogenetic analysis using quantitative, high sensitivity, fluorescence hybridization. *Proc Natl Acad Sci* **83**: 2934–2938

Prescher G, Bornfeld N and Becher R (1990) Nonrandom chromosomal abnormalities in primary uveal melanoma. *J Natl Cancer I* **82**: 1765–1769

Robinson JK, Rigel DS and Amonette RA (1997) Trends in sun exposure knowledge, attitudes, and behaviors: 1986–1996. *J Am Acad Dermatol* **37**: 179–186

Ross DA and Wilson GD (1998) Expression of c-myc oncoprotein represents a new prognostic marker in cutaneous melanoma. *Brit J Surg* **85**: 46–51

Schlagbauer-Wadl H, Griffioen M, von Elsas A et al (1999) Influence of Increased c-Myc Expression on the Growth Characteristics of Human Melanoma. *J Invest Dermatol* **112**: 332–336

Sisley K, Rennie IG, Parsons MA, Jacques R, Hammond DW, Bell SM, Potter AM and Rees RC (1997) Abnormalities of chromosomes 3 and 8 in

 *© 2001 Cancer Research Campaign*

posterior uveal melanoma correlate with prognosis. *Gene Chromosome Canc* **19**: 22–28

Taub R, Kirsch I, Morton C, Lenoir G, Swan D, Tronick S, Aaronon S and Leder P (1982) Translocation of the c-myc gene into the immunoglobulin heavy chain locus in human Burkitt lymphoma and murine plasmacytoma cells. *Proc Nat Acad Sci* **79**: 7837–7841

Thompson EB (1998) The many roles of c-myc in apoptosis. *Annu Rev Physiol* **60**: 575–600

Visscher DW, Wallis T, Awussah S, Mohamed A and Crissman JD (1997) Evaluation of myc and chromosome 8 copy number in breast carcinoma by interphase cytogenetics. *Gene Chromosome Canc* **18**: 1–7

Vogelstein B and Kinzler KW (1993) The multistep nature of cancer. *Trends Genet* **9**: 138–141

*© 2001 Cancer Research Campaign*
